# Regulation of renal aquaporins: implications in tubular epithelial integrity

**DOI:** 10.1042/BST20253114

**Published:** 2026-01-12

**Authors:** Vishalini Venkatesan, Charlotte M. Sørensen, Emma Tina B. Olesen

**Affiliations:** 1 Department of Biomedical Sciences, University of Copenhagen, Denmark

**Keywords:** cell migration, oxidative stress, tubular injury, water reabsorption

## Abstract

Aquaporins (AQPs) are crucial membrane proteins that primarily facilitate water transport across cell membranes. In the kidneys, AQP1, AQP7, AQP8, and AQP11 are expressed in the proximal tubules. AQP1 is also localized to the thin descending limb of the loop of Henle. AQP2, AQP3, AQP4, AQP5, and AQP6 are expressed in the collecting ducts. Specific AQPs, such as aquaglyceroporins and peroxiporins, also transport solutes like glycerol and hydrogen peroxide, indicating their broader physiological roles beyond water permeability. Renal AQPs play a fundamental role in urine concentration and maintaining water balance. However, some studies using AQP knockout mouse models have reported structural abnormalities in the renal tubules, along with defective water handling. These findings highlight the involvement of AQPs in regulating cell proliferation, migration, and apoptosis, which are essential processes for maintaining tubular integrity. Furthermore, aquaglyceroporins and peroxiporins are implicated in modulating cellular redox balance and contributing to oxidative stress responses that are also associated with tubular damage. This review explores how AQPs are regulated under physiological conditions and how they become dysregulated in kidney diseases such as acute kidney injury, diabetic kidney disease, and polycystic kidney disease. Understanding these mechanisms may help in identifying new therapeutic strategies targeting AQPs in renal pathologies.

## Introduction

Tubular reabsorption of electrolytes and water constitutes a major part of total renal oxygen consumption. This process occurs across the renal tubular epithelial cells (RTECs) that are the fundamental units of tubular architecture. RTECs preserve structural integrity by maintaining apicobasal polarity, supporting both transcellular and paracellular transport of solutes and fluids. Transcellular water reabsorption is critical for maintaining fluid balance and occurs via water channels, termed aquaporins (AQPs). Several isoforms of these channels are expressed in RTECs across different segments of the renal tubules. These channels in the plasma membrane mediate selective transcellular water transport in response to osmotic gradients. AQPs function as homotetramers, with each monomer composed of six membrane-spanning ɑ-helices that serve as water channels [1]. Nine AQP isoforms (AQP1-8 and AQP11) are expressed in RTECs across various tubular segments, with five of them (AQP1-4 and AQP7) being important for water transport [2]. Specific AQPs have distinct regulatory mechanisms that will be discussed later in this review.

Beyond their well-characterized role as water transporters, AQPs are also involved in key processes essential for tubular repair post-injury, including cellular migration, proliferation, differentiation, and apoptosis. AQPs, by allowing water transport, regulate the cell volume. Alterations in cell volume induce the formation of lamellipodia and filopodia that then depend on actin polymerization and cytoskeletal rearrangements for promoting cell migration [3,4]. Moreover, the theoretical ‘osmotic engine model’ explains that localization of specific AQPs and ion channels in the leading and lagging ends of the polarized RTECs, and the consequent water and ion fluxes aid in propelling the cell forward [4]. Expression of AQPs is closely associated with mitogen-activated protein kinase (MAPK) signaling pathways that are also involved in transcription of protein machinery required for cell proliferation, differentiation, and apoptosis [5–7]. In renal diseases that involve loss of tubular integrity, such as acute kidney injury (AKI), autosomal dominant polycystic kidney disease (ADPKD), and diabetic nephropathy (DN) [8–10], damage to RTECs is observed along with dysregulation of AQPs [11]. In this mini-review, we summarize how major water channels in RTECs are regulated during tubular reabsorption and kidney diseases.

## AQPs in the Proximal Tubule - AQP1, AQP7, AQP8 and AQP11

### AQP1

**Figure 1:**
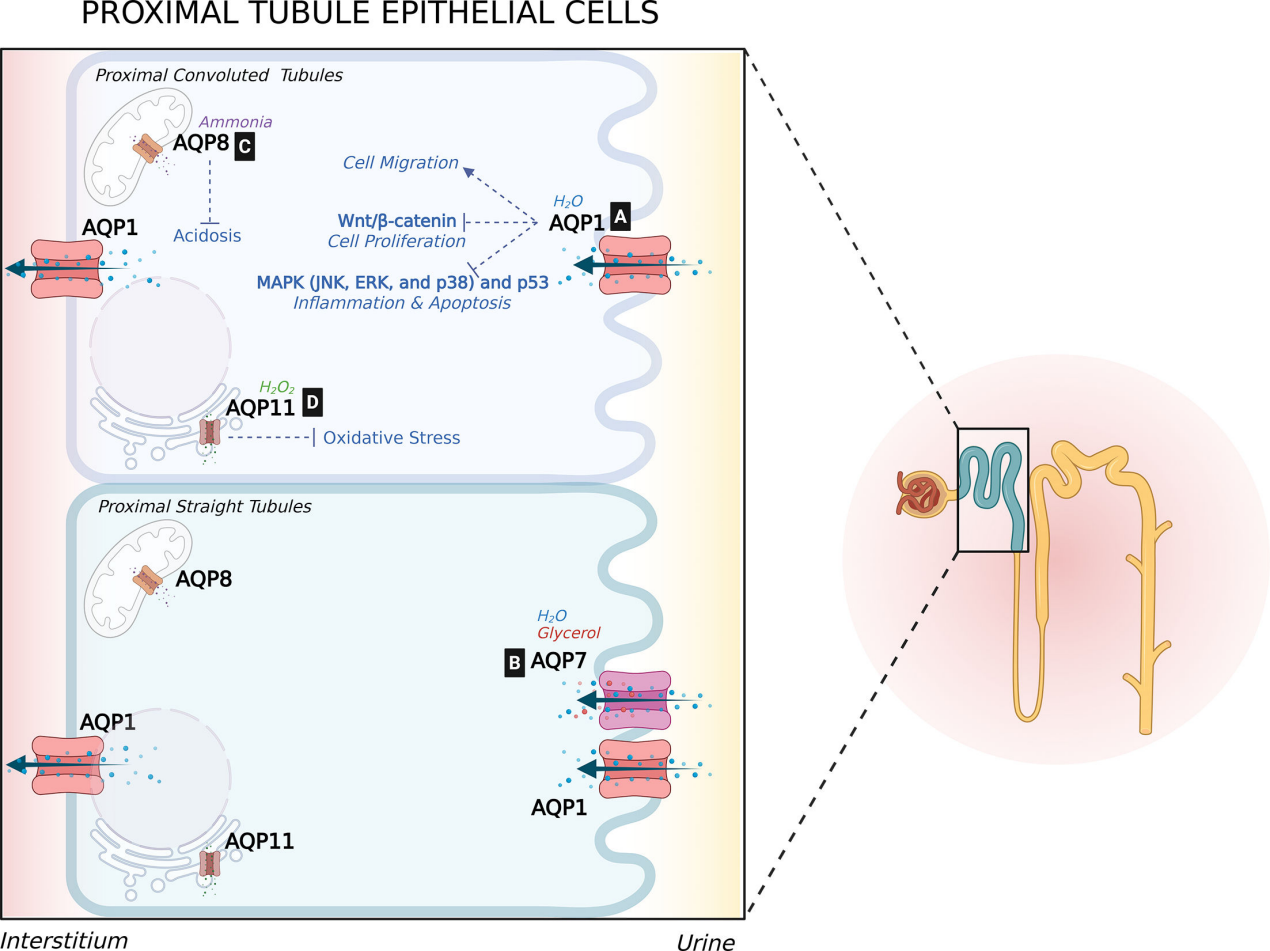
Figure 1 Role of proximal tubule AQPs in tubular transport and architecture. This illustration highlights the location of AQPs in proximal tubule epithelial cells and their roles in tubular transport and integrity. [**A**] AQP1 is present on both the apical and basolateral membranes, where it supports tubular repair by promoting cell migration and reducing inflammation through inhibition of p53 and MAPK signaling. It is also renoprotective in ADPKD, as it suppresses Wnt/β-catenin signaling, thereby reducing abnormal cell proliferation. [**B**] AQP7 is expressed in the brush border apical membrane of the proximal straight tubule, where it primarily transports glycerol and, to a lesser extent, water. [**C**] AQP8 is localized to the inner mitochondrial membrane and helps adapt to metabolic acidosis by regulating ammonia transport. [**D**] AQP11 is located in the endoplasmic reticulum and controls intracellular H₂O₂ levels, contributing to oxidative stress regulation. *Illustration created with BioRender.com.* p53, tumor protein 53; MAPK, mitogen-activated protein kinase; JNK, Jun N-terminal kinase; ERK, extracellular signal-regulated kinase; H₂O₂, hydrogen peroxide.

The proximal convoluted tubule (PCT) reabsorbs approximately 65% of the water from the glomerular filtrate. Water is transported across the RTECs via AQP1 channels that are expressed abundantly on both the apical and basolateral sides (Figure 1A) [12]. Mice lacking AQP1 show markedly reduced water reabsorption in the proximal tubule and the descending thin limb of the loop of Henle. This impairs counter-current multiplication and leads to a severe urinary concentrating defect [12,13]. Humans with the AQP1 mutation also exhibit a mild urine concentrating defect, which is evident only after water deprivation [14]. In a recent study in streptozotocin (STZ)-induced diabetic rats, AQP1 mRNA and protein levels initially increased at 15 days. However, after 5 months, AQP1 protein levels decreased, coinciding with tubular dilation and glomerular alterations. This also corroborated with concentrating defects, increased creatinine clearance, and proteinuria. Thus, AQP1 down-regulation was suggested to exacerbate osmotic diuresis in the model [15]. AQP1 is constitutively expressed and not acutely regulated at the transcriptional level by hormones [15]. Acute regulation of AQP1 membrane targeting is not widely accepted. But cAMP-dependent protein kinase A (PKA) enhances AQP1 phosphorylation in rat kidney homogenates, although the exact phosphorylation site remains unidentified [16]. The functional consequences of this phosphorylation remain unknown.

In addition to its role in transepithelial water transport, AQP1 is essential for proximal tubule cell migration, which is vital for maintaining PCT integrity, and its down-regulation is even considered a marker of proximal tubule injury [17–19]. AQP1 is proposed to facilitate water entry into lamellipodia at the leading edge, promoting polarity in migrating human endothelial and melanoma cell lines [20]. This mechanism has not been studied in renal epithelial cells; however, primary proximal tubule cells from AQP1 null mice show more than a 50% decrease in cell migration compared with wildtype (WT) cells [21]. In addition, ischemic injury in AQP1-null mice resulted in significantly greater proximal tubule dilation, accompanied by abnormal actin organization, compared with WT mice [21]. This finding highlights the protective role of AQP1 in kidney injury. Importantly, this may not solely be due to tubular AQP1 expression. AQP1 also plays an important role in inflammatory responses by promoting macrophage polarization toward the M2 phenotype, which supports wound-healing processes [22]. During lipopolysaccharide (LPS)-induced AKI, AQP1 promotes the phosphoinositide 3-kinase (PI3K) pathway or inhibits the P38 MAPK pathway that consequently promotes M2 polarization and alleviates injury [22,23]. Transcriptomic analysis of renal tissue from septic AKI mice and blood samples from AKI patients revealed reduced AQP1 expression and increased p53 levels [24]. In septic AKI rats, AQP1 silencing up-regulated p53 expression and inflammatory factors (Tumor necrosis factor-α (TNF-α), interleukin-1β (IL-1β), interleukin-6 (IL-6)and pNF-κB), which elevated apoptosis and fibrosis, exacerbating kidney damage. Conversely, AQP1 overexpression reversed these effects, reducing inflammation and injury in LPS-induced AKI and HK-2 cells (human kidney-2 cells; immortalized human proximal tubule epithelial cell line) [24]. Another study also reported that in LPS-induced HK-2 cells, overexpression of AQP1 inhibited MAPK p38, extracellular signal-regulated kinase 1/2 (ERK1/2), and Jun N-terminal kinase (JNK) signaling, reducing inflammation and increasing cell viability [7]. In a rat model of LPS-induced AKI, the angiotensin-II receptor blocker irbesartan reduced inflammation as measured by reduced NF-κB levels, and in parallel, AQP1 expression was restored and kidney function was improved [17]. Questions remain on how this effect was mediated in the model, where both inflammatory and hemodynamic mechanisms could be in play.

The role of AQP1 in maintaining RTEC integrity is further supported by findings in AQP1-deficient Madin-Darby canine kidney (MDCK) cells and polycystin-1 (Pkd1) knockout (KO) mice [25]. In these models, AQP1 deficiency led to cyst development, which was primarily driven by activation of the Wnt signaling pathways. Supporting this, MDCK cells overexpressing AQP1 (AQP1-MDCK) showed reduced proliferation and cell adhesion. In 3D cyst culture, these cells exhibited a 3.2-fold increase in tubulogenesis. However, when grown on transwells, AQP1-MDCK and control cells showed no difference in water permeability after forskolin stimulation, suggesting this effect is unrelated to AQP1’s water transport function [25]. These findings suggest the protective role of AQP1 in preserving epithelial integrity and alleviating cyst formation during ADPKD. Altogether, these findings underscore the multifaceted role of AQP1 in proximal tubular function beyond its canonical role in water transport. From mediating cellular migration and injury repair to modulating inflammatory and fibrotic responses, AQP1 emerges as a critical determinant of proximal tubular integrity and a potential therapeutic target in kidney disease.

### AQP7

The proximal straight tubule contains an abundance of the aquaglyceroporin AQP7 localized on the brush border apical membrane. AQP7 primarily facilitates glycerol transport and, to a lesser degree, water transport from the tubular fluid into the interstitium (Figure 1B) [26]. In AQP7 KO mice, proximal tubular water permeability was lower, whereas glycosuria was significantly greater compared with WT controls. AQP1/AQP7 double KO mice display a more severe urinary concentrating defect at baseline than AQP1 KO mice, indicating the contribution of AQP7 in the proximal tubule water resorption [27]. The urinary glycerol concentration in AQP1/AQP7 double KO mice is 2.4 mM, accounting for only a small portion of the total urine osmolality (600 mOsm/kg H₂O), indicating that the concentrating defect is not primarily due to osmotic diuresis. It is estimated that AQP7 contributes only about one-eighth as much as AQP1 to water permeability in the proximal tubules [28]. Western blotting and Quantitative Reverse Transcription Polymerase Chain Reaction (qRT-PCR) studies displayed no compensatory increase in AQP7 expression in AQP1 KO mice, proposing that AQP7 is primarily regulated by glycerol levels rather than water balance [28]. Studies did not report any significant changes in PCT structures, suggesting that AQP7 may not contribute notably to maintaining tubular structure [28]. There is a lack of studies to determine how AQP7 is regulated during kidney injury and its potential role in maintaining tubular epithelial integrity.

### AQP8

AQP8 is expressed in the inner mitochondrial membrane of rat proximal tubules and mildly in the collecting ducts [29] (Figure 1C), and has been shown to regulate ammonia transport to adapt to metabolic acidosis in HK-2 cells and rats [30]. AQP8-null mice exhibit normal baseline urine-concentrating ability and ammonia excretion, and no tubular damage has been reported, making its role in maintaining tubular structure unclear [31].

### AQP11

Superaquaporin AQP11 is expressed in the endoplasmic reticulum (ER) of the proximal tubule epithelial cells (Figure 1D). Although its role in tubular water transport remains unknown, AQP11 has a major role in maintaining kidney tubular integrity. It transports hydrogen peroxide (H_2_O_2_) from the ER lumen to cytosol and regulates intracellular H_2_O_2_ concentration and ER stress. AQP11 is critical during kidney development, whereas its physiological role in adult kidneys appears limited [32]. Kidney-specific loss of AQP11 causes a form of polycystic kidney disease due to the importance of H_2_O_2_ for the biogenesis of polycystin-1 [33]. Although AQP11 evidently contributes to maintaining tubular architecture in the developing kidney, its role in ADPKD is likely limited, as the disease is primarily caused by mutations in Pkd1 or Pkd2. However, a case of renal cystic disease was recently described due to AQP11 mutations [34]. In addition, the AQP11 rs2276415 variant has been linked to the development of kidney disease in type 2 diabetes [35] and the progression of chronic kidney disease in the absence of risk factors [36], making the pathophysiological role of AQP11 an interesting avenue for further investigation.

## AQPs in the Collecting Duct – AQP2, AQP3, AQP4, AQP5 and AQP6

**Figure 2:**
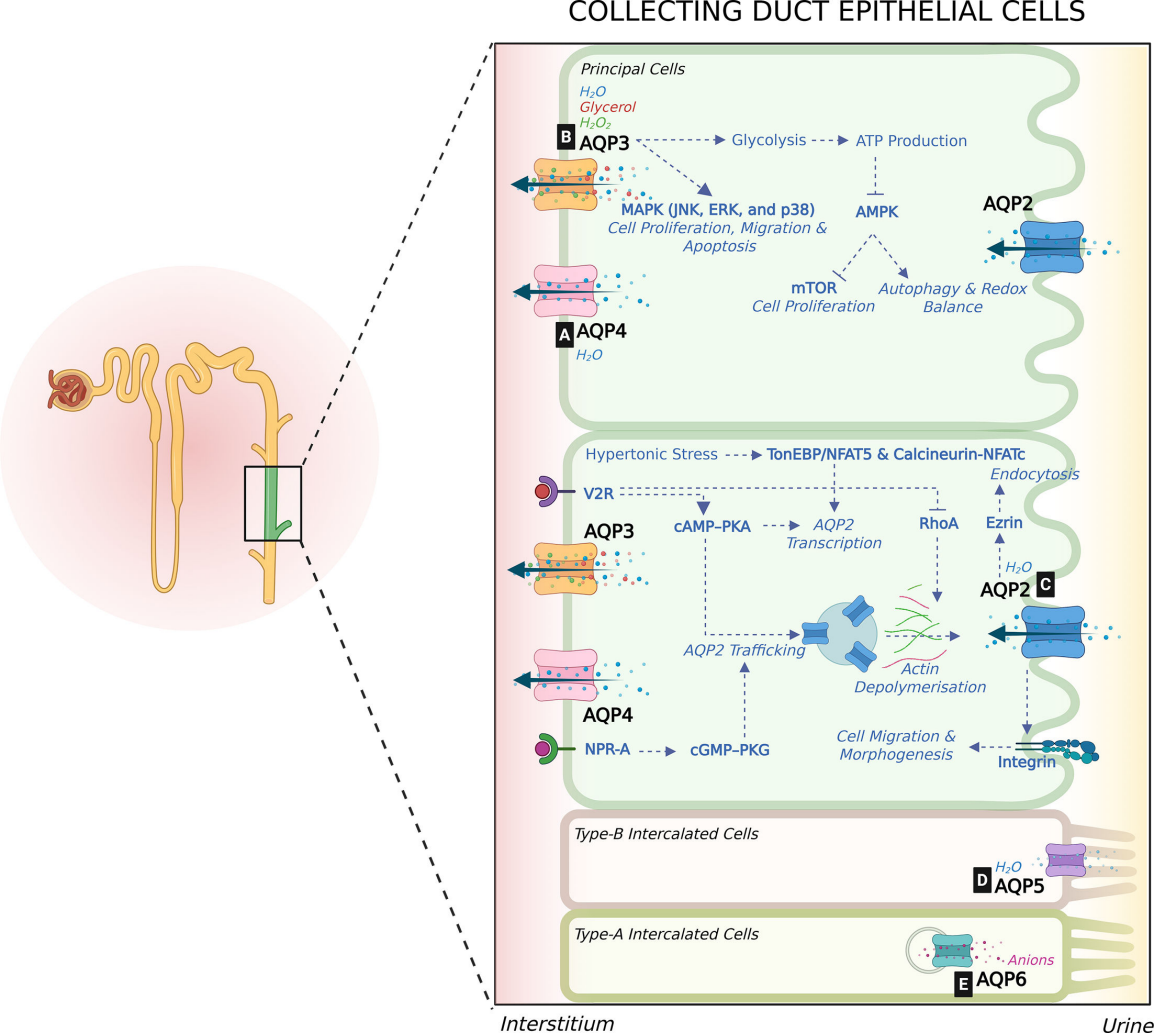
Regulation of collecting duct AQPs and their role in tubular transport and structure. This illustration summarizes the regulatory mechanisms of AQPs in collecting duct epithelial cells and their roles in water transport and tubular integrity. In principal cells, water transport is mediated by AQP3 and AQP4 on the basolateral membrane and AQP2 on the apical membrane. [**A**] The role of AQP4 in tubular integrity remains unclear. [**B**] In addition to water, AQP3 transports glycerol and H₂O₂, which enhance glycolysis and ATP production, thereby worsening polycystic kidney disease. Elevated ATP levels inhibit the AMPK pathway, leading to increased mTOR-driven cell proliferation. By facilitating H₂O₂ flux, AQP3 also regulates redox balance and autophagy. However, during kidney injury, AQP3 supports tubular repair by promoting cell proliferation, migration, and apoptosis through MAPK pathway activation. [**C**] AQP2 is stored in intracellular vesicles and translocates to the apical membrane upon V2R stimulation, which also increases its transcription. AQP2 is further regulated by hypertonic stress via TonEBP/NFAT5 and calcineurin–NFATc signaling, as well as by ANP and NO through the cGMP–PKG pathway. RhoA inhibition facilitates apical trafficking by inducing actin depolymerization. AQP2 interacts with ezrin to mediate endocytosis. AQP2 through integrin β1 promotes cell migration and morphogenesis. [**D&E**] In type B intercalated cells, AQP5 is localized to the apical membrane and mediates water transport, while in type A intercalated cells, AQP6 is found in intracellular vesicles, where it regulates anion transport and helps maintain acid–base balance. *Illustration was created using Biorender.com.* H₂O₂, hydrogen peroxide; MAPK, mitogen-activated protein kinase; JNK, c-Jun N-terminal kinase; ERK, extracellular signal-regulated kinase; ATP, adenosine triphosphate; AMPK, AMP-activated protein kinase; mTOR, mammalian target of rapamycin; TonEBP/NFAT5, tonicity-responsive enhancer binding protein/nuclear factor of activated T-cells 5; V2R, vasopressin type 2 receptor; cAMP, cyclic adenosine 3',5'-monophosphate; PKA, protein kinase A; RhoA, ras homolog family member A; NPR-A, natriuretic peptide receptor A; cGMP, cyclic guanosine monophosphate; PKG, protein kinase G.

In the collecting duct (CD), water reabsorption and urine concentration are intricately regulated by AQP2, AQP3, and AQP4 in principal cells. AQP2 is stored in intracellular vesicles. When vasopressin activates vasopressin type 2 receptor (V2R) signaling, AQP2 is inserted into the apical membrane, enabling water transport. AQP3 and AQP4 are stably expressed on the basolateral membrane and are not acutely regulated by hormone receptors. However, long-term vasopressin treatment increases AQP3 expression [37]. AQP4 KO mice displayed only mild concentrating defects upon water deprivation and showed no abnormal kidney morphology, implying that AQP4 may not play a significant role in maintaining tubular integrity or water homeostasis [38] (Figure 2A).

### AQP3

In addition to water transport, AQP3 also mediates glycerol and H₂O₂ flux, playing a key role in oxidative stress regulation (Figure 2B). Glycerol is metabolized through glycolysis and tricarboxylic acid cycle, generating adenosine triphosphate (ATP) alongside reactive oxygen species such as H₂O₂. While H₂O₂ can cause oxidative damage, it also acts as a secondary messenger in various protein kinase signaling pathways that regulate cell autophagy, proliferation, and migration [39]. Therefore, by fluxing glycerol and H₂O₂, AQP3 may regulate the cell’s redox balance. AQP3 KO mice exhibit renal insufficiency, as demonstrated by elevated blood urea nitrogen and creatinine levels compared with WT mice [6]. While concentrating defects may cause these elevations, AQP3 KO mice also showed loss of CD integrity, characterized by CD dilation and hyperplasia in both sham and ischemia-reperfusion-operated models. These mice also exhibit increased markers of oxidative stress and inflammation, indicating AQP3’s renoprotective role during kidney injury. Tubular regeneration following injury requires cell proliferation, migration, and reduced apoptosis [6]. Studies in MDCK cells have shown that AQP3 overexpression via MAPK signaling significantly enhances cell proliferation and migration while reducing apoptosis, highlighting its role in tubular regeneration [6].

In contrast to the renoprotective role in AKI, AQP3 has been implicated in promoting CD cell proliferation and cyst enlargement in ADPKD, both *in vivo* using AQP3-null PKD mice and *in vitro* using MDCK cells stably expressing human AQP3. Increased glycolysis and ATP generation have been observed in Pkd1-deficient mouse models, contributing to cyst progression [40]. AQP3-mediated glycerol uptake increases ATP production, inhibits AMP-activated protein kinase (AMPK) activity, and activates both extracellular signal-regulated kinase (ERK) and mammalian target of rapamycin (mTOR) pathways, collectively driving cell proliferation[1] [41]. Moreover, the permeability of AQP3 to H₂O₂ may further enhance glycolysis [42] and accelerate cyst growth. Substantiating this mechanism, AQP3 deletion reduced cyst size and slowed cyst progression in an ADPKD mouse model [41]. In STZ-induced diabetic mice, concentrating defects coincided with reduced AQP3 expression in the renal cortex, while medullary CD expression remained unchanged. There was a concurrent reduction in AQP2 expression, but other underlying mechanisms have not been investigated, particularly as the study did not report any tubular damage [43]. In summary, AQP3 plays a dual role in kidney health. While it promotes cyst enlargement in ADPKD via metabolic and signaling pathways, it also offers renoprotective benefits in kidney injury models by managing oxidative stress. These findings place AQP3 as a novel potential drug target in kidney diseases where tubular damage is a key driver.

### AQP2

AQP2 is expressed in the principal cells of the CDs and is stored in intracellular vesicles (Figure 2C). Vasopressin binding to V2R triggers both short-term and long-term regulation of AQP2. Long-term regulation involves transcription and translation of the AQP2 gene following vasopressin stimulation. Short-term regulation occurs via translocation of AQP2 from intracellular vesicles to the apical membrane. This process is initiated by V2R activation, which elevates intracellular cAMP and activates PKA. Mouse AQP2 is phosphorylated at four serine residues (S256, S264, S269, and S261) in the carboxy terminal, with phosphorylation of S256 essential for AQP2 exocytosis. Interestingly, S256 phosphorylation and AQP2 membrane targeting are not exclusively dependent on cAMP and PKA [44]. Nitric oxide donors and atrial natriuretic peptide can also induce S256 phosphorylation by increasing cGMP and activating PKG, thereby promoting AQP2 trafficking to the plasma membrane [45]. After V2R activation, phosphorylation of S264 and S269 also increases [46], although neither phosphorylation site is required for AQP2 membrane targeting [47]. AQP2 phosphorylated at S261 decreased with vasopressin stimulation and was mostly found to be localized to the sub-apical cellular space, unlike pS256-AQP2, which was strongly associated with the apical membrane [48,49]. Taken together, this indicates that pS261-AQP2 is linked to endocytosis and ubiquitination. While ubiquitination at the K270 lysine residue directs AQP2 to endosomal compartments for recycling or degradation, pS261-AQP2 is suggested to stabilize the ubiquitinylated AQP2 [50]. Besides V2R activation, the activation of E-prostanoid receptors EP2 and EP4 has been shown to increase phosphorylation of AQP2-S256 and AQP2-S264 and increase apical AQP2 localization [51]. It has also been shown that after blocking Rho GTPase, which regulates actin polymerization and prevents AQP2 translocation, apical AQP2 and water permeability increases even without vasopressin stimulation [52–55]. AQP2 is also shown to interact directly with an actin-binding protein, ezrin, to facilitate clathrin-mediated endocytosis [56]. AQP2 expression increases under hyperosmotic conditions via activation of tonicity-responsive enhancer binding protein/nuclear factor of activated T-cells 5 (TonEBP/NFAT5) and calcineurin-NFATc pathways in both murine principal kidney cortical collecting duct and murine inner medullary collecting duct (mIMCD) cells [57–59]. Novel mechanisms of AQP2 regulation have been extensively reviewed [44].

In addition to its intricate role in regulating water homeostasis, AQP2 may play a significant role in maintaining tubular integrity. AQP2 KO mice have growth retardation, postnatal mortality, and severe urinary concentrating defects [60]. In addition, these mice develop abnormally dilated tubules and microcysts, primarily originating from CDs. This underscores AQP2’s role in maintaining tubular structure and function and suggests its potential contribution to cyst progression in polycystic kidney disease [44]. In a mouse model with CD-specific inactivation of hepatocyte nuclear factor-1β (HNF-1β), cystic kidneys developed along with a urinary concentrating defect, despite elevated cAMP levels and AQP2 protein expression. However, AQP2 accumulated in the cytosol and basolateral regions instead of localizing to the apical membrane, indicating a trafficking defect in cystic kidney disease. Interestingly, the concentrating defect was observed even at the pre-cystic stage, indicating that it was due to the AQP2 trafficking disorder rather than the presence of tubular cysts. HNF-1β mutant kidneys have decreased collectrin that is involved in the vesicular trafficking of proteins [61]. The most common form of polycystic kidney disease is caused by Pkd1 inactivation, and in pre-cystic nephron-specific Pkd1 KO mice, no differences in AQP2 expression were observed when compared with control mice [62]. The regulation of AQP2 during cyst progression remains unexplored. It has been shown that AQP2 interacts with a cell adhesion molecule, integrin β1, via the second external loop RGD (arginine–glycine–aspartic acid [Arg–Gly–Asp]) motif to regulate epithelial cell migration and morphogenesis, but not cell proliferation and apoptosis. Both MDCK and porcine proximal tubule (porcine kidney epithelial cell line [LLC-PK1]) cells transfected with AQP2 have enhanced cell migration in a wound healing assay and formed tubules in 3D culture compared with the cells with the mutated RGD motif that instead formed cyst structures. The same study has also established that the promigratory effects of AQP2 are not directly influenced by the water permeability of AQP2 water channels in the epithelial cells [63]. AQP2-expressing cells have smaller focal adhesions because of rapid water flux. This speeds up cell migration but is not critical for it [64]. Pyroptosis, a form of cell death associated with inflammation, was reduced with AQP2 overexpression in HK-2 cells, likely due to the down-regulation of caspase-1 expression. However, it is important to note that HK-2 cells, which are from the proximal tubule, may not fully represent AQP2 regulation in the CDs [65]. Conversely, in apoptotic rat cortical CD cells, AQP2 expression increased both caspase-1 levels and the rate of apoptosis [66]. It remains uncertain whether and how AQP2 is directly related to inflammation in kidneys.

### Integrated roles of AQP2, AQP3 and AQP4 in CD epithelial integrity

While previous studies examined individual CD water channels, one study explored the combined role of AQP2, AQP3, and AQP4 in maintaining CD integrity by regulating cell migration in cultured primary IMCD cells. Cell migration increased with an acute rise in medium osmolality and vice versa. This was also investigated using a wound healing assay in IMCD cells, which endogenously express all CD AQPs, allowing analysis of their co-ordinated regulation. During cell migration under hyperosmotic conditions and PKA activation, AQP2 was observed to localize at the trailing edge, while AQP4 was found at the leading edge. As the water enters through the leading edge and exits through the trailing edge, the cells are propelled forward according to the theoretical osmotic engine model. Additionally, AQP3 expression was absent from the first few rows of the leading edge, suggesting that AQP3 does not actively contribute to cell migration [67]. Thus, although AQP3 overexpression in MDCK cells has been shown to enhance cell migration in scratch-wound and transwell migration assays [6], AQP3 may not have a direct role in cell migration in IMCD cells [67]. AQP 2–3 KO studies in IMCD cells may elucidate the precise role of each of the AQPs in CD cell migration. Also, the role of AQP4 during AKI has not been investigated in KO mice. Collectively, it is possible that AQP2 and AQP4 play a role in tubular integrity after injury through their roles in cell migration, whereas the role of AQP3 in maintaining normal cellular metabolism is essential for maintenance of tubular structure.

### AQP5 and AQP6

Other AQPs in the CDs include AQP5 that is localized on the apical membrane of type B intercalated cells [68] and AQP6 that is found within the intracellular vesicles of type A intercalated cells [69] (Figure 2D and E). Although AQP6 has been proposed to participate in renal acid-base regulation [70], its physiological role is not characterized. AQP5 has been implicated in diabetic kidney disease, where its expression is up-regulated and positively correlates with disease severity, suggesting its potential use as a biomarker of tubular dysfunction [71]. AQP5 has also been shown to colocalize with AQP2 in the perinuclear region, resulting in reduced apical AQP2 expression and contributing to polyuria [72]. However, currently, there is no evidence indicating a role for AQP5 or AQP6 in the maintenance of tubular epithelial architecture.

## Conclusion

Alongside water reabsorption, some AQPs also contribute to cell migration and tissue repair [Table 1], making them promising yet challenging therapeutic targets in kidney disease [80]. While direct AQP2 modulators show potential for treating water balance disorders, none have reached clinical use due to key challenges: (1) multiple AQP isoforms share conserved structures, complicating isoform-selective drug design; (2) lack of robust functional assays to validate AQP-targeting drugs and measure water, glycerol, and H₂O₂ permeability; (3) widespread AQP expression across tissues, increasing off-target risks; and (4) incomplete understanding of mechanistic roles for some AQPs [80,81]. Nevertheless, AQPs are currently used as biomarkers of kidney injury and may even help to localize damage to specific nephron segments based on urinary AQP profiles [82,83]. Altogether, although much is currently known about the highly diverse roles of AQPs in the kidney, further mechanistic insights are needed, especially for proximal tubule AQPs and their potential roles in tubular integrity. Recently, molecular dynamics simulations have advanced our understanding of AQP structural dynamics and gating mechanisms by simulating complex environments like renal epithelia [84]. Coupled with high-throughput screening, these advances may enable discovery of novel AQP modulators and clarify their roles in tubular integrity [85].

**Table 1:** Overview of kidney aquaporin isoforms, including their locations, functions, regulatory mechanisms, abnormal kidney histology reported in KO mice, and related pathological roles.

Type of AQPs	Location	Function	Regulation	Kidney histology in KO mice	Pathological role
AQP1	Apical and basolateral plasma membrane of proximal tubules and DTL [15]	Water permeability [12]; cell migration, inflammatory and fibrotic responses [21,24]		Dilated PCT [21]	Urinary concentrating defect in AQP1 KO mice [12]
AQP2	Stored in intracellular vesicles and trafficked to apical plasma membrane of collecting ducts upon V2R stimulation [73]	Water permeability [73]; cell migration and morphogenesis [63,67]	Canonical V2R signaling pathway, cAMP–PKA-independent pathways include A-kinase anchoring protein (AKAP)–PKA disruptors, Src (a tyrosine-protein kinase) inhibition, Wnt5a, fluconazole, and epidermal growth factor receptor (EGFR) antagonists [44]	Tubular dilation and microcyst formation in neonatal mice [63]	AQP2 trafficking disorder and urinary concentrating defect in the CD–specific HNF-1β KO mouse, a cystic kidney disease model [61]
AQP3	Basolateral plasma membrane of principal cells in collecting ducts [74]	Water, glycerol, and H_2_O_2_ permeability; oxidative stress [6]	Long-term V2R stimulation [74]	Collecting duct dilation and hyperplasia [6]	NDI in AQP3 KO mice [75]; Enhances kidney cyst growth in Pkd1 KO mice [41]
AQP4	Basolateral plasma membrane of principal cells in collecting ducts [76]	Water permeability [76]; cell migration [67]		No abnormal morphology [38]	Mild concentrating defect upon water deprivation in AQP4 KO mice [38]
AQP5	Apical plasma membrane of type B intercalated cells in collecting ducts [68]	Negative regulator of apical AQP2 localization [72]			Urine AQP5/creatinine is elevated in DN [71,77]
AQP6	Intracellular vesicles of type A intercalated cells in collecting ducts [69]	Renal acid-base regulation [70]			
AQP7	Apical plasma membrane of proximal straight tubules [26]	Glycerol and water permeability [28]			
AQP8	Inner mitochondrial membrane of proximal tubules [29]	Mitochondrial ammonia transport [30]			
AQP11	ER of proximal tubules [78]	H_2_O_2_ permeability [32]; managing oxidative stress [78]		Polycystic proximal tubules and ER vacuolization [79]	Polycystic kidneys in AQP11 KO mice [79] and humans [34]; associated with diabetic kidney disease [35,36]

**Perspectives**

- **Highlight importance of the field:** In kidney diseases such as acute kidney injury, diabetic nephropathy, and autosomal dominant polycystic kidney disease, water balance disorders often occur alongside tubular injury. Both processes involve dysregulation of aquaporins (AQPs), proposing these channels as potential therapeutic targets that could address both aspects of disease pathology.
- **Summary of the current thinking:** Since the discovery of AQP1 in 1992 as a water channel, extensive research has characterized multiple AQP isoforms, uncovering their regulatory mechanisms and diverse physiological roles beyond water permeability, particularly AQP2. Despite their established role in kidney pathophysiology, therapeutic targeting remains limited due to the absence of selective and effective modulators.
- **Comment on future directions:** Novel small molecules targeting specific AQPs are emerging and may enhance current treatments for kidney diseases. Future work should evaluate their therapeutic potential, either as monotherapies or in combination with existing drugs, with an emphasis on improving specificity and efficacy.

## Abbreviations

ADPKD: autosomal dominant polycystic kidney disease
AKAP: A-kinase anchoring protein
AKI: acute kidney injury
AMPK: AMP-activated protein kinase
AQPs: aquaporins
ATP: adenosine triphosphate
CD: collecting duct
DN: diabetic nephropathy
ER: endoplasmic reticulum
ERK: extracellular signal-regulated kinase
HK-2: human kidney-2 cells (immortalized proximal tubule epithelial cell line)
HNF-1 β: hepatocyte nuclear factor 1 β
H_2_O_2_: hydrogen peroxide
JNK: Jun N-terminal kinase
KO: knockout
LLC-PK1: porcine kidney epithelial cell line (from pig proximal tubule)
LPS: lipopolysaccharide
MAPK: mitogen-activated protein kinase
MDCK: Madin-Darby canine kidney (cell line)
NPR-A: natriuretic peptide receptor A
PCT: proximal convoluted tubule
PI3K: phosphoinositide 3-kinase
PKA: protein kinase A
PKG: protein kinase G
Pkd1: polycystin-1
RGD: arginine–glycine–aspartic acid (peptide motif)
RTECs: renal tubular epithelial cells
RhoA: ras homolog family member A
STZ: streptozotocin
TNF-α: tumor necrosis factor-α
TonEBP/NFAT5: tonicity-responsive enhancer binding protein/nuclear factor of activated T-cells 5
V2R: vasopressin type 2 receptor
WT: wildtype
cAMP: cyclic adenosine 3',5'-monophosphate
cGMP: cyclic guanosine monophosphate
mIMCD: murine inner medullary collecting duct
mTOR: mammalian target of rapamycin
